# Conjoint analysis of transcriptome and metabolome profiles of normal captivity and arch soil free-range in Meishan pigs

**DOI:** 10.3389/fvets.2023.1187877

**Published:** 2023-07-27

**Authors:** Ying Liu, Yanlong Su, Zhijie Zhou, Jie Zhu, Qianqian Zhu, Peng Xie, Shiquan Qian, Liwei Wang, Tong Qin, Gang Zhou

**Affiliations:** ^1^School of Life Science, Huaiyin Normal University, Huaian, China; ^2^Huaiyin Institute of Agricultural Sciences in Xuhuai Region, Huaian, China; ^3^Institute of Animal Sciences, Chinese Academy of Agricultural Sciences, Beijing, China

**Keywords:** Meishan pigs, transcriptomic, metabolomic, normal captivity, arch soil

## Abstract

The hygiene hypothesis has been advanced as a potential explanation for the increasingly high levels of atopy and allergic disease in the general human population. In an effort to conduct a more detailed study of the link between immune activity and the hygiene hypothesis, Meishan pigs raised under normal captivity (NC) or arch soil free-range (ASF) conditions were selected as an experimental model system. Cytokine levels were found to differ significantly between these two groups consistent with a difference in cellular immune status. Integrated transcriptomic and metabolomic profiling of duodenal tissue samples from Meishan pigs were then performed, leading to the identification of differentially expressed genes (DEGs), differentially abundant metabolites (DAMs), and key pathways that were able to distinguish the NC and ASF groups. This approach led to the identification of 1,113 DEGs, as well as 577 and 372 DAMs in positive and negative ion modes, respectively. When an interaction network incorporating DEGs and metabolites associated with immune responsivity was constructed, it included factors such as 9-cis-Retinoic acid, (9Z,11E)-(13S)-13-Hydroxyoctadeca-9,11-dienoic acid and (10E,12Z)-(9S)-9-Hydroxyoctadeca-10,12-dienoic acid. Functional enrichment analyses confirmed that identified DEGs and DAMs were associated with immune-related pathways including the intestinal IgA production and PPAR signaling pathways. Together, these results offer new insight into the roles that particular genes and metabolites enriched in response to environmental stressors in free-range Meishan pigs may play in the regulation of cellular immunity, thus offering a foundation for future efforts to better understand the immunological mechanisms underlying the hygiene hypothesis.

## Introduction

The so-called hygiene hypothesis is a model that posits that reduced exposure to less sanitary conditions and infections during early life may increase the future risk of atopy and allergic disease. An early study performed by Braun-Fahrländer et al. (1) determined that children raised in rural settings were exposed to higher levels of microorganisms in their daily lives, and were also less likely to develop allergies or asthma, with these results having subsequently been confirmed repeatedly throughout the globe (2). However, asthma and inflammatory bowel disease rates in certain lower-income nations have remained fairly low for reasons that remain to be clearly established (3). The hygiene hypothesis provides an attractive explanation for these observations such that it has been the subject of increasing public and scientific attention (4). When the innate immune system encounters bacteria or other microorganisms, they can cause an inflammatory response even when there is no obvious infection (5). The presence of some level of microbes and microbial byproducts in the environment where an individual is raised is thus conducive to the development of a more mature and robust immune system, while also reducing the odds of developing allergies in response to potential environmental allergens. The hygiene hypothesis, while initially observed in humans, also holds true in other species. Wild boars that reside in the mountains, for example, exhibit more robust immunity than do domestic pigs (6). Microbial abundance in the surrounding environment is thus an important regulator of mammalian immune system development. More detailed studies of the mechanisms underlying this relationship have the potential to help alleviate the societal burden imposed by allergic diseases and related conditions, offering fundamentally novel opportunities to help control or prevent autoimmune diseases while ensuring healthy immunological development.

To data, more studies in the hygiene hypothesis showed that the composition of gut microbiome is vital to the regulation of health and disease in humans (7, 8). The composition of the microflora is now thought to be a major mediator of this model of immunological development, with the degree of microbial exposure or deprivation serving as a key parameter (9). It is therefore vital to more fully explore the association between environmental microbial abundance and the establishment of a robust immune system. Free-living and captive porcine populations are highly adaptable to a range of environmental conditions (10), potentially contributing to differences in intestinal immunity. In this study, Meishan pigs raised under normal captivity (NC) or arch soil free-range (ASF) conditions were selected as a model system to explore the immunological basis for the hygiene hypothesis through analyses of cytokine levels in these animals. A conjoint analysis of the transcriptomic and metabolomic profiles of pigs from these NC and ASF groups was further conducted to better explore the ability of environmental factors to influence gene expression and metabolite composition.

## Materials and methods

### Ethics statement

This study received approval from the Animal Experiments Ethics Committee and Institutional Animal Care and Use Committee (IACUC) of Huaiyin Normal University, with all procedures involving animals having been conducted as per the Regulations for the Administration of Affairs Concerning Experimental Animals approved by the State Council of the People’s Republic of China.

### Animal care

In total, 40 weaning Meishan piglets of similar body shape and weight were obtained from Kunshan Conservation Ltd. (Suzhou City, Jiangsu Province, China). These pigs were randomly assigned to two equally sized groups including the normal captivity (NC) and arch soil free-range (ASF) groups. Animals in the NC group were housed in a climate-controlled room in four pens and were provided a standard swine diet and raised under matching husbandry conditions. Pigs in the ASF group were transferred to a soil-containing yard at 09:00 each day and were allowed to roam freely, subsequently being returned to their pens at 17:00. All pigs in both groups were fed identical grower diets.

### Sample collection

On days 1 and 30, jugular vein blood samples were collected from all experimental animals. Blood samples were centrifuged to isolate serum, which was then snap-frozen and stored at −60°C for subsequent cytokine analyses. On day 30, 4 piglets per group of similar body shape and weight were selected and euthanized via intravenous pentobarbital sodium injection to minimize suffering. The duodenum was then resected from each of these piglets, snap-frozen using liquid nitrogen, and subsequently used for metabolomic and transcriptomic analyses.

### Cytokine measurements

A range of different cytokines (GM-CSF, IFN-γ, TNF-α, IL-2, IL-1α, IL-1β, IL-1ra, IL-10, IL-6, IL-8, IL-4, IL-12 and IL-18) were detected in serum samples from 8 pigs per group using a PCYTMG-23 K-13PX Premixed Kit (Merck, Germany) based on provided directions using a Luminex X-200 instrument at Novogene Bioinformatics Technology Co. Ltd. (Beijing, China).

### Metabolomic sample processing

Duodenum tissues between NC group (*n* = 6) and ASF group (*n* = 6) were used for metabolomic analyses. The extraction and identification of metabolites and the processing of associated data from LC–MS analyses were performed by Genedenovo Tech Co., Ltd. (Guangzhou, China). Briefly, equal amounts of samples were lyophilized, suspended in 1,000 uL of cold (−20°C) methanol, centrifuged (10 min, 12,000 rpm, 4°C), and 450 μL supernatant aliquots were concentrated under vacuum before dissolution in 150 μL of 80% methanol supplemented with 2-chlorobenzalanine (4 ppm) prior to filtration with a 0.22 μm membrane. Additionally, 20 μL aliquots from each sample were used to conduct quality control (QC) analyses, with the remainder being utilized for LC–MS analyses. A Vanquish UHPLC system with an Orbitrap Q ExactiveTM HF-X mass spectrometer (Thermo Fisher, Germany) was employed to conduct UHPLC–MS/MS analyses.

### Metabolite identification

Raw data were processed as in a prior report (11). Briefly, Proteowizard (v 3.0.8789) converted these raw data files into the mzXML format, after which peak identification, filtration, and alignment were conducted with the R (v3.3.2) XCMS package (12) using the following settings: bw = 5, ppm = 15, peakwidth = c (5,30), mzwid = 0.01, mzdiff = 0.01, method = “centWave.” Subsequent analyses were then performed based on the m/z ratio, retention time, intensity, and positive/negative precursor ions. Batch normalization of peak intensity to the total spectral intensity was performed. Metabolites were identified based on exact molecular formulae (error < 20 ppm), with peaks then being matched using Metlin^1^ and MoNA^2^ to confirm metabolite annotations.

### Transcriptomic sequencing

Trizol (Invitrogen, CA, United States) was used for RNA extraction in duodenum tissues between NC group (*n* = 4) and ASF group (*n* = 4) based on provided directions, after which an Agilent 2,100 Bioanalyzer (Agilent Technologies, CA, United States) and agarose gel electrophoresis were employed to assess RNA quality. The RIN values were within the range of 7.4 ~ 8.2. Oligo(dT) beads were then used for eukaryotic mRNA enrichment, with a Ribo-Zero™ Magnetic Kit (Epicentre, WI, United States) being used for rRNA removal. Enriched mRNA samples were suspended in a fragmentation buffer to generate short fragments, after which cDNA was generated via reverse transcription using random primers. DNA polymerase I, RNase H, and dNTPs were then employed for second-strand cDNA synthesis, followed by the use of a QiaQuick PCR extraction kit (Qiagen, Venlo, Netherlands) to purify this cDNA. After isolation, cDNA was subjected to end repair, poly-adenylation, and Illumina sequencing adapter ligation. Agarose gel electrophoresis was then used for ligation product size selection, with subsequent PCR amplification and sequencing with the Illumina HiSeq2500 platform performed by Gene Denovo Biotechnology Co. (Guangzhou, China).

### Transcriptomic data analyses

Reads were filtered using fastp (v 0.18.0) to isolate high-quality reads (13). Any rRNA reads mapped to the rRNA database using the Bowtie 2 short read alignment tool (v 2.2.8) (14) (version 2.2.8) were removed from the dataset, while the remaining clean reads were retained for analysis. A reference genome index was etablished, with HISAT 2.2.4 being used for the mapping of clean paired-end clean reads to the Sscrofa11.1 genome assembly^3^ (15) using “-rna-strandness RF” and other default parameters. A reference-based approach was used for assembling mapped reads with StringTie v1.3.1 (16, 17). FPKM values were computed with StringTie as a means of calculating relative gene expression, and DESeq2 was used to compare differential gene expression between groups (18), while differential expression between samples was compared with edgeR (19). Those genes that exhibited a false discovery rate (FDR) < 0.05 as well as an absolute fold change ≥2 were regarded as differentially expressed genes (DEGs). These DEGs were arranged into volcano plots and subjected to Gene Ontology (GO) (20) and Kyoto Encyclopedia of Genes and Genomes (KEGG) pathway (21) enrichment analyses.

### Transcriptomic and metabolomic association analyses

DEGs identified above were integrated with differentially abundant metabolites (DAMs) to identify overlapping metabolic pathways using a two-way orthogonal partial least-squares (O2PLS) approach to detect any immune-related pathways represented in both the DEG and DAM datasets. Metabolomic and transcriptomic data were also subjected to integrated analysis with Pearson correlation coefficients, and pairs of genes and metabolites in shared pathways exhibiting a Pearson correlation coefficient > 0.995 and a *p*-value <0.05 were visualized with Cytoscape (V3.3.0). A metabolite transcript network analysis was performed with the top 250 gene-metabolite pairs exhibiting a Pearson correlation coefficient > 0.5 using R graphing packages.

### qPCR validation

Selected DEGs were validated using a qPCR approach using primers synthesized by Invitrogen Biotechnology Co., Ltd. (Shanghai, China) as compiled in Supplementary Table S1. To ensure the specificity of amplification reactions, standard curves and melt curves were generated for all primer pairs. *GAPDH* served as a reference control gene. Each 20 μL qPCR reaction contained 10 μL of SYBR^®^ Premix Ex Taq (Tli RNaseH Plus) (2×) (TaKaRa Biotechnology Co., Ltd., Dalian, China), 0.4 μL of forward and reverse primers, 0.4 μL of ROX Reference Dye (50×) (TaKaRa), 2 μL of cDNA, and 6.8 μL of distilled water. Thermocycler settings were 95°C for 30 s; 40 cycles of 95°C for 5 s, 60°C for 30 s. Relative gene expression was assessed via the 2^−ΔΔCt^ method. Samples were analyzed in triplicate and averaged together to quantify gene expression.

### Statistical analysis

SPSS 18.0 software (IBM Corp., Armonk, NY, United States) was used. Relative quantitative PCR results were examined via the 2^−ΔΔCt^ method. Between groups comparisons were made using Student’s *t* test. Data represents the mean ± SD. Differences were significant statistically at *p* < 0.05 or *p* < 0.01.

## Results

### Differences of cytokine levels in Meishan pigs between NC and ASF groups

**Figure 1:**
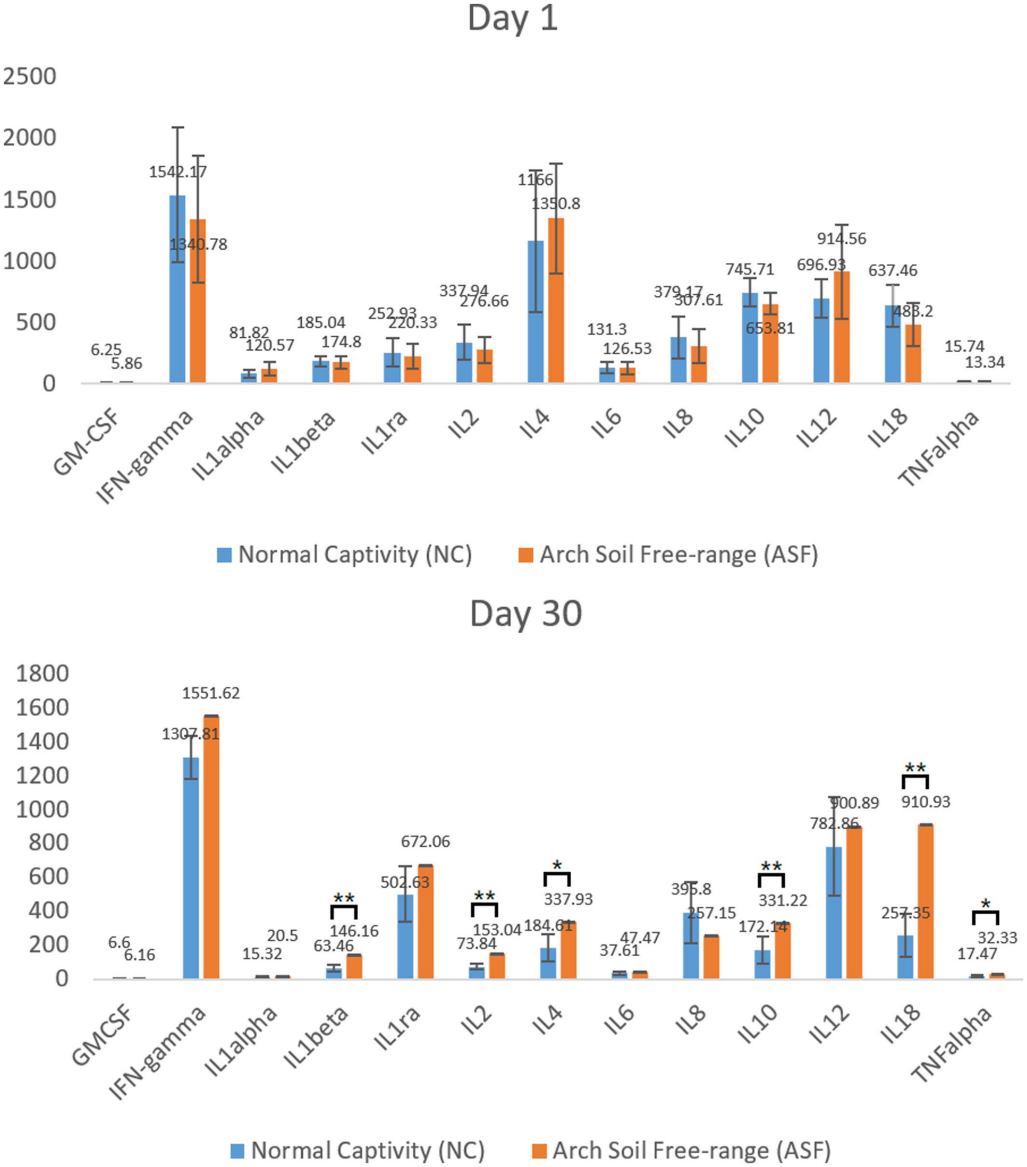
Analysis of serum cytokine levels in the normal captivity (NC) group and arch soil free-range (ASF) groups on days 1 and 30. Data are means±SD, ^*^p < 0.05, ^**^p < 0.01.

To explore differences in the immune function between the NC and ASF groups, an initial analysis of serum inflammatory cytokine levels was conducted on days 1 and 30 to gauge the cellular immunity status in these animals. No significant differences in analyzed cytokine levels were observed between groups on day 1 (Figure 1A). On day 30, however, levels of IL-1β, IL-2, IL-10, TNF-α, IL-4, and IL-18 were significantly increased in the ASF group relative to the NC group (*p* < 0.05), with other cytokines other than IL-8 and GM-CSF also exhibiting a trend towards increased expression in the ASF group (Figure 1B, *p* > 0.05). These changes suggest a significant difference in the cellular immune status of pigs in the ASF and NC groups.

### Transcriptome analyses screened out DEGs between NC and ASF groups

**Figure 2:**
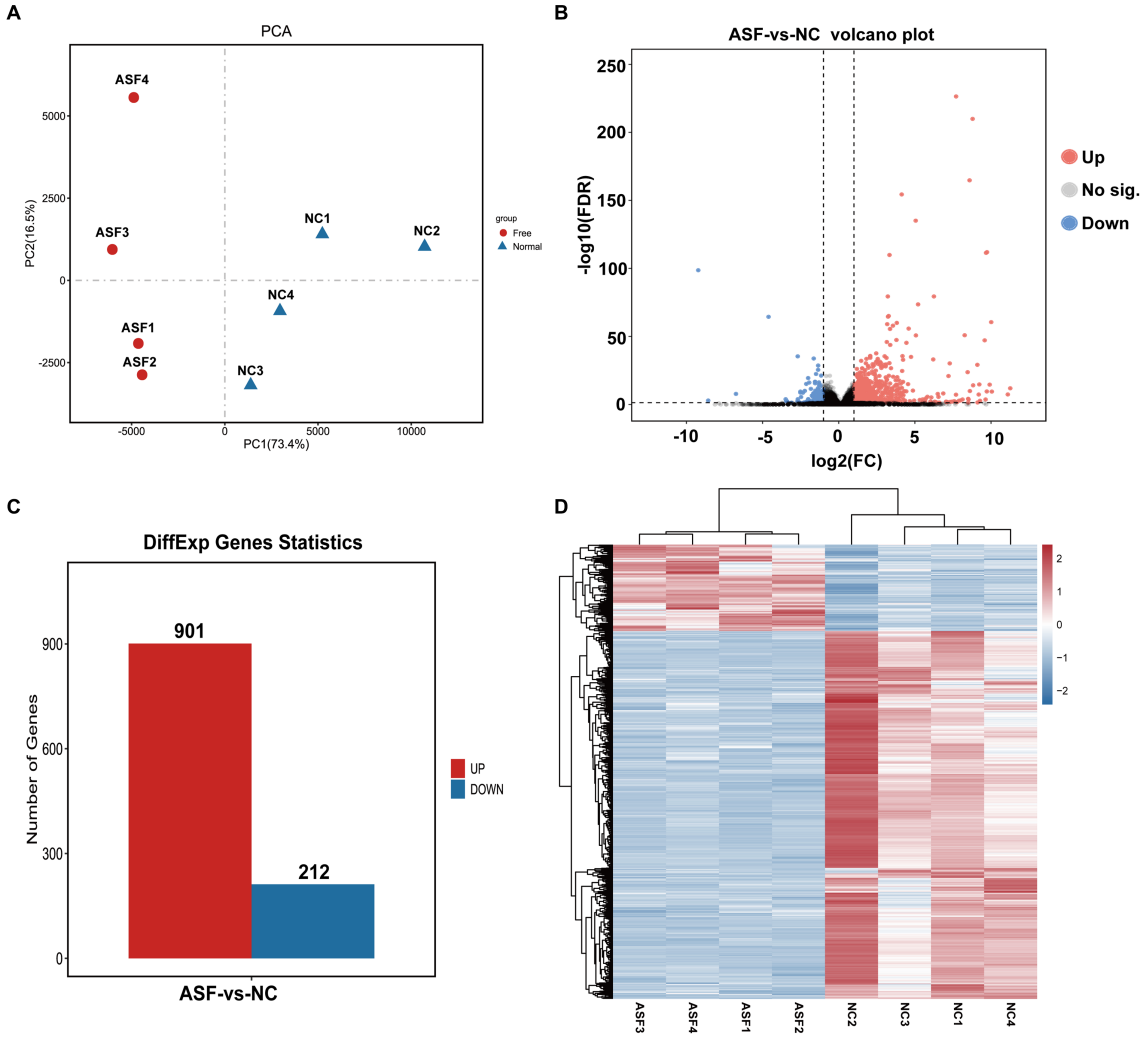
Transcriptomic analysis results for duodenal samples from pigs in the NC and ASF groups. **(A)** PCA analysis of samples from the NC and ASF groups. **(B)** Differences in gene expression between the two groups were represented with a volcano plot. **(C)** A representation of the numbers of DEGs that were upregulated and downregulated when comparing the NC and ASF groups. **(D)** Heatmap analysis of DEGs identified in the 8 analyzed sequencing libraries, with upregulated and downregulated genes, respectively, shown in red and blue.

Next, four duodenal tissue samples from each group (ASF and NC) were used to prepare RNA libraries that were sequenced with the Illumina HiSeq 2,500 platform. In total, 2720.6 and 2809.4 million raw reads were obtained for the ASF and NC libraries, respectively, with 2708.5 and 2798.6 million clean reads remaining after filtering was complete (Supplementary Table S2). A principal component analysis (PCA) of the clean data revealed good repeatability for these samples (Figure 2A), making them well-suited to downstream analysis. A total of 15,795 genes expressed in the duodenum of pigs were detected in ASF group and NC group. DEGs were then identified based on standard criteria (log2(FC) > 1, *p* < 0.05) (Figure 2B), leading to the identification of 1,113 total DEGs of including 901 that were upregulated (80.95%) and 212 that were downregulated (19.05%) in the ASF group (Supplementary Table S3; Figure 2C), including the top 10 DEGs, such as *SULT1E1, CUBN, SLC13A1, SPP1, XPNPEP2, TMIGD1, FABP6, SLC1A1, LOC100526118, GCG*. A heatmap-based comparison of DEG expression profiles in these two groups revealed that DEGs in the ASF and NC samples clustered together separately from one another, with the replicates in each group clustering well with one another (Figure 2D).

**Figure 3:**
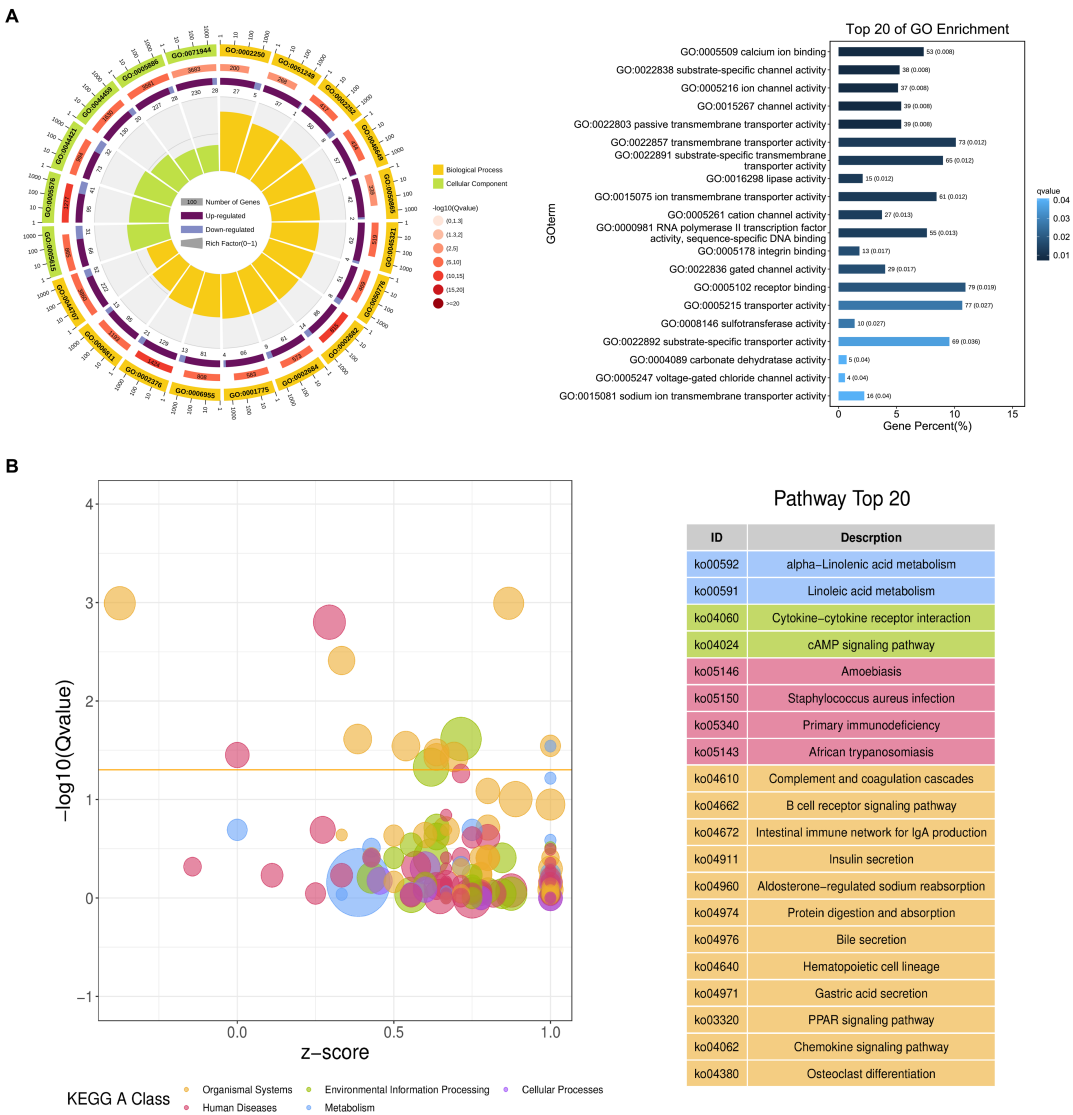
Functional annotation of DEGs identified when comparing duodenal samples from the NC and ASF groups. **(A)** Gene ontology (GO) enrichment analysis results highlighting GO terms from the biological process (BP) and cellular component (CC) categories. **(B)** A bubble diagram highlighting differences in KEGG pathway enrichment. Z-scores are the ratio of the difference between the numbers of upregulated genes and downregulated genes to the total number of DEGs, with a yellow line at the Q-value threshold of 0.05.

The possible functions of identified DEGs were explored through GO and KEGG enrichment analyses. Most of these DEGs were associated with GO terms such as “transmembrane transporter activity,” “receptor binding,” and “transporter activity” (Figure 3A). These DEGs were also significantly enriched in 20 KEGG pathways (*p* < 0.05) (Supplementary Table S4; Figure 3B), including the “Intestinal immune network for IgA production (ko04672)” and “PPAR signaling (ko03320)” pathways which may function as important regulators of immune functionality.

### Metabolomic analysis screened out DAMs between between NC and ASF groups

**Figure 4:**
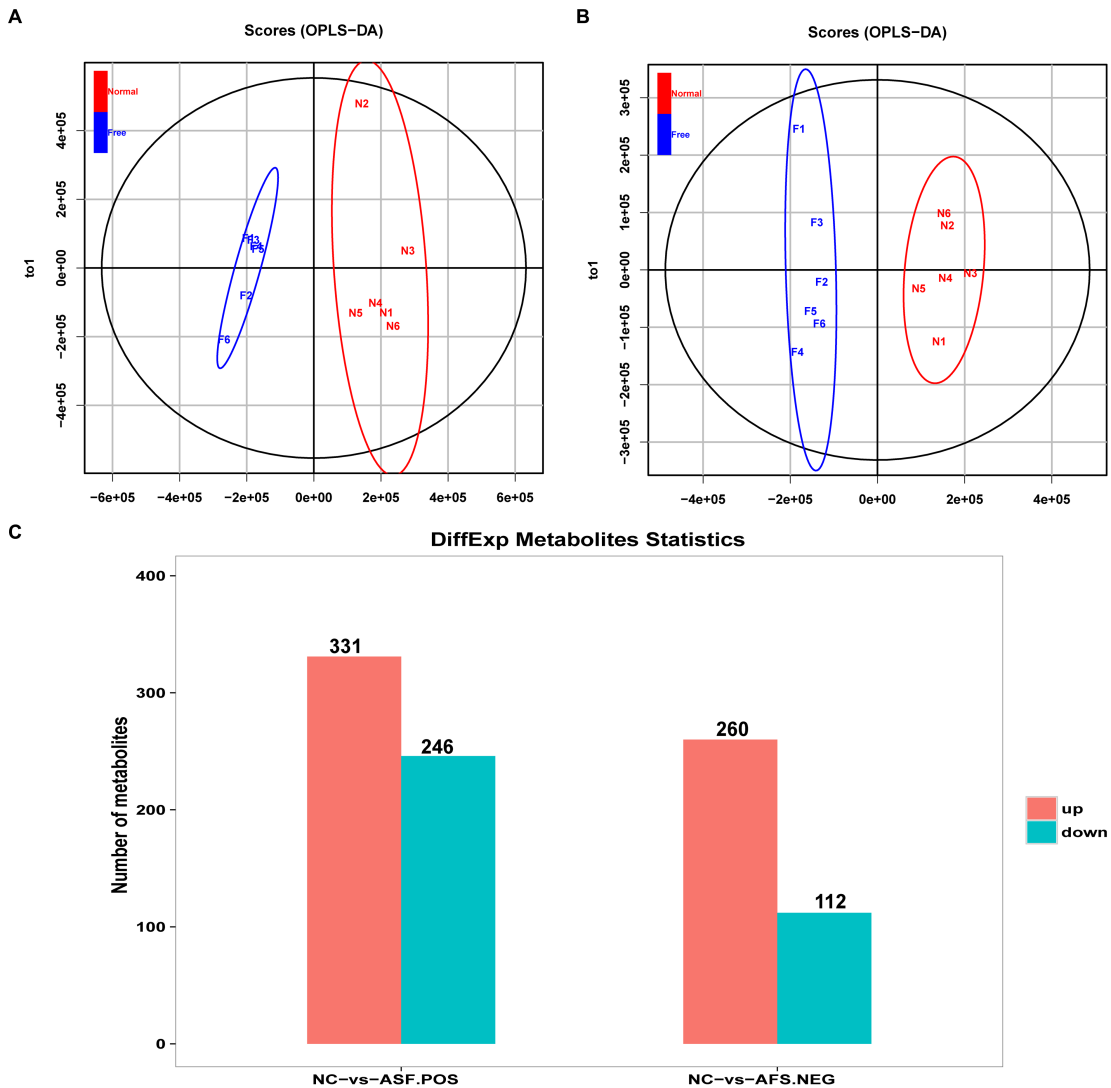
Analysis of metabolites differentially abundant between the NC and ASF groups. **(A,B)** OPLS-DA score plots for polar metabolites identified in duodenal metabolic profiling analyses. Data were derived from LC–MS profiles for the two groups in positive (POS, A) and negative (NEG, B) ion modes. **(C)** A histogram highlighting the number of DAMs identified when comparing the NC and ASF groups.

**Figure 5:**
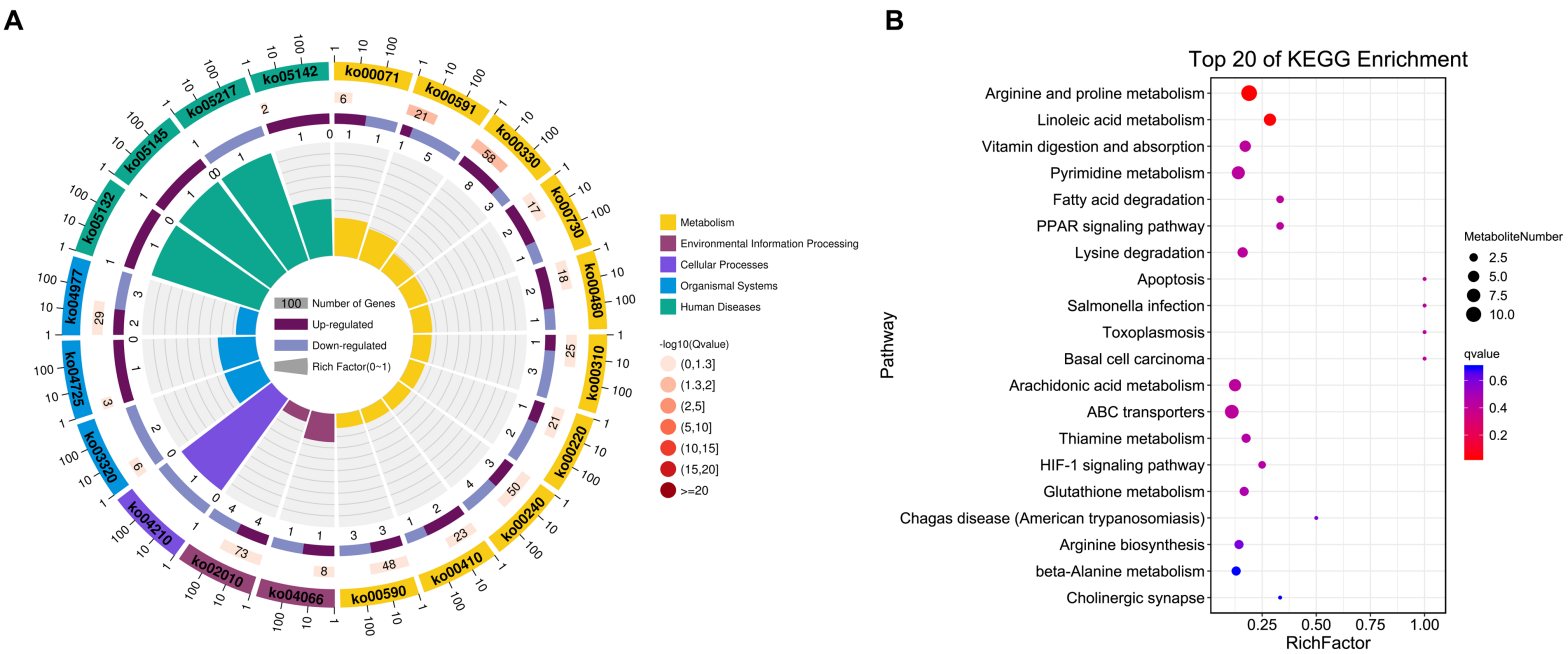
KEGG enrichment results for identified DAMs. **(A)** DAM-related pathway distributions. **(B)** KEGG enrichment analysis results for DAMs, with the pathway name shown along the y-axis and the degree of enrichment along the x-axis. The size of the circles corresponds to the number of included DAMs, while the color is proportional to the associated *q*-value.

An untargeted metabolomics approach was next used to explore the potential relationship between rearing conditions and metabolomic changes in the duodenum of these experimental pigs. Samples from the NC and ASF groups were separated clearly on a PCA plot (Figures 4A,B). A total of 10,593, 16,591 annotated metabolites were identified in positive ion (POS) and negative ion (NEG) modes, respectively. OPLS-DA analysis revealed clear differences between the two groups indicating that their metabolite profiles differed significantly, with a total of 577 (331 up- and 246 downregulated) differentially abundant metabolites (DAMs) in NC group compared to ASF group based on positive ion (POS) mode, including the top 10 DAMs, such as N-Acetylmuramate, N,N-Dimethylsphingosine, Sebacic acid, 5,6-DHET, 4-Aminophenol, Pyrroline hydroxycarboxylic acid, 4-Hydroxy-5-phenyltetrahydro-1,3-oxazin-2-one, Spermidine, Prostaglandin-c2, Dulcin (Figure 4C; Supplementary Table S5). Besides, a total of 372 (260 up- and 112 downregulated) DAMs were also identified based on negative ion (NEG) mode, including the top 10 DAMs, such as Flumioxazin, Toluate, Ascorbate, 13S-hydroxyoctadecadienoic acid, Deoxyguanosine, alpha-Ketoisovaleric acid, 9(S)-HPODE, Actinonin, Fexofenadine, 3-Hydroxybenzoic acid (Figure 4C; Supplementary Table S6). having been identified when comparing the ASF and NC groups in positive and negative ion modes (Figure 4C; Supplementary Tables S5, S6). Following metabolite identification, KEGG enrichment analysis indicated that the majority of these metabolites were significantly enriched in immune-related signaling pathways including the PPAR signaling and Salmonella infection pathways (Figure 5).

### Conjoint analysis of metabolomic and transcriptomic identified key immune-related genes between NC and ASF groups

**Figure 6:**
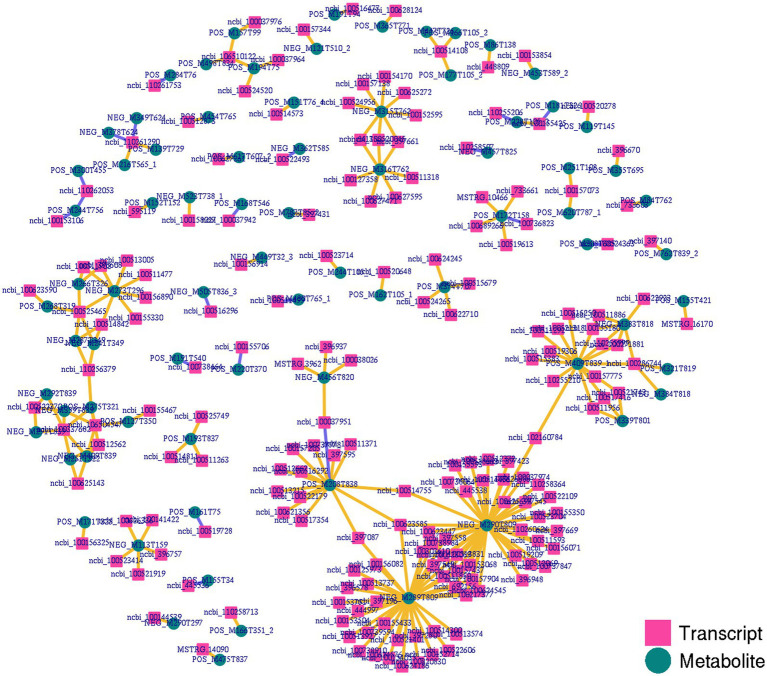
Integrated network analyses of DEGs and DAMs. Transcripts and metabolites are, respectively, represented with squares and circles, highlighting co-expression network results for DEGs and DAMs identified when comparing the NC and ASF groups.

**Figure 7:**
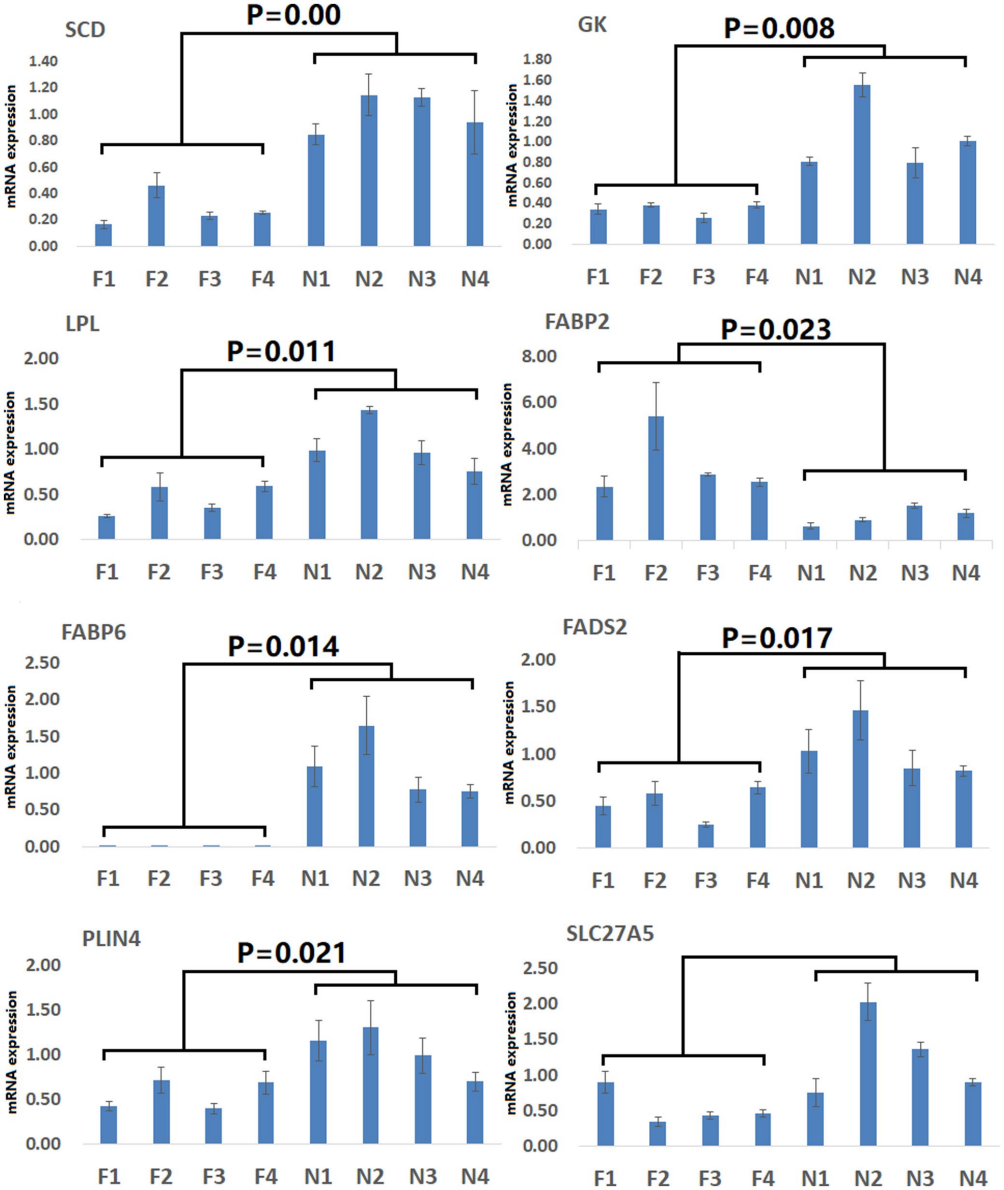
Validation of PPAR signaling pathway-related DEGs. Validation was performed via qPCR. F1–F4: pigs (*n* = 4) from arch soil free-range (ASF) group; N1–N4: pigs (*n* = 4) from normal captivity (NC) group. Data are means±SD.

**Figure 8:**
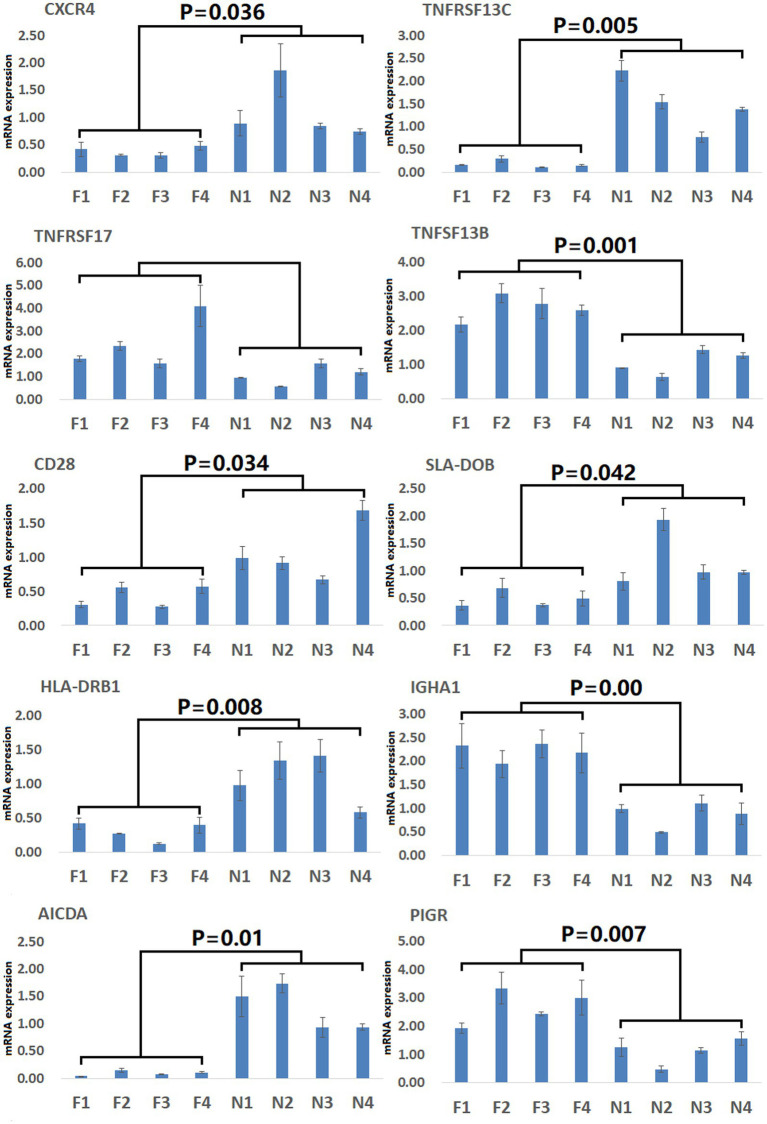
Validation of Intestinal immune network for IgA production pathway-related DEGs. Validation was performed via qPCR. F1–F4: pigs (*n* = 4) from arch soil free-range (ASF) group. N1–N4: pigs (*n* = 4) from normal captivity (NC) group. Data are means±SD.

To gain more detailed insights into the relationships between identified DEGs and DAMs, we constructed the regulatory network between genes and metabolites (Figure 6), enabling the analysis of potential regulatory gene-metabolite pairs identified in the duodenal samples of pigs when comparing the NC and ASF groups. This analysis led to the identification of 10 shared KEGG pathways for these DEGs and DAMs (Table 1), two of which were immunological pathways including the Intestinal immune network for IgA production and PPAR signaling pathways. A qPCR-based approach was used to compare the expression of PPAR signaling-related genes (*SCD, GK, LPL, FABP2, FABP6, FADS2, SLC27A5, PLIN4*) between duodenal samples from the NC and ASF groups (Figure 7). Higher levels of *SCD, GK, LPL, FABP6, FADS2,* and *PLIN4* expression were evident in the NC group (*p* < 0.05 or *p* < 0.01), whereas *FABP2* was expressed at higher levels in the ASF group (*p* < 0.05), and *SLC27A5* levels were comparable in these two groups. Similarly, a qPCR approach was used to validate the observed differences in the expression levels of DEGs related to the Intestinal immune network for IgA production pathway (Figure 8), confirming significant differences in *TNFRSF13C, TNFSF13B, IGHA1, PIGR*, *CXCR4, CD28, SLA-DOB,* and *AICDA* expression when comparing the two groups (*p* < 0.05 or *p* < 0.01), while *TNFRSF17* expression did not differ significantly between ASF and NC pig samples.

**Table 1:** Integration analysis of KEGG annotations for DEGs and DAMs identified when comparing the normal captivity (NC) and arch soil free-range (ASF) groups.

Pathway	Gene NC-*vs*-ASF	Metabolite NC-*vs*-ASF	Pathway ID	Genes	Metabolites
Amoebiasis	17	1	ko05146	ncbi_100037951(C9);ncbi_100037953(C8A);ncbi_396859(NOS2);ncbi_100628112(IL1R2);ncbi_100154994(SERPINB10);ncbi_397115(ARG1);ncbi_397208(CSF2);ncbi_399500(IL6);ncbi_100518131(PIK3CD);ncbi_397620(FN1);ncbi_100157958(LAMC3);ncbi_100736677(GNAL);ncbi_100623759(LAMB4);ncbi_100522792(PRKCB);ncbi_100625589(LAMA1);ncbi_100157641(PLCB2);MSTRG.15983(IGHA1)	POS_M175T87
Protein digestion and absorption	13	4	ko04974	ncbi_445538(XPNPEP2);ncbi_448809(COL10A1);ncbi_541593(FXYD2);ncbi_397532(COL5A2);ncbi_397003(SLC1A1);ncbi_396828(ATP1A2);ncbi_396892(PGA5);ncbi_100154136(MEP1A);ncbi_100155319(COL9A1);ncbi_100516141(MEP1B);ncbi_100520915(COL11A2);ncbi_641346(SLC6A19);ncbi_100519955(COL22A1)	POS_M102T325;POS_M147T81_1;POS_M175T87;NEG_M164T332_2
Bile secretion	11	5	ko04976	ncbi_541593(FXYD2);ncbi_396828(ATP1A2);ncbi_100512372(SLC51A);ncbi_100625109(SLC10A2);ncbi_100153960(NR1H4);ncbi_396910(ABCB1);ncbi_397073(ABCG2);ncbi_397639(EPHX1);ncbi_100526226(NR0B2);ncbi_100153359(LOC100153359);ncbi_102158419(SLC27A5)	POS_M104T89_1;POS_M145T836;POS_M369T816;NEG_M392T738;NEG_M501T816_1
Linoleic acid metabolism	6	6	ko00591	ncbi_100512662(PLA2G2D);ncbi_100513737(PLA2G2F);ncbi_396971(ALOX15);ncbi_100514108(PLA2G5);ncbi_100519306(PLB1);ncbi_100514811(PLA2G4E)	POS_M279T800_2;POS_M297T736;NEG_M293T825;NEG_M295T832;NEG_M311T796;NEG_M329T662
PPAR signaling pathway	10	2	ko03320	;ncbi_595106(FABP2);ncbi_100233182(GK);ncbi_444997(FADS2);ncbi_396670(SCD);ncbi_397423(FABP6);ncbi_397537(LPL);ncbi_100524667(PLIN4);ncbi_102158419(SLC27A5)	(9Z,11E)-(13S)-13-Hydroxyoctadeca-9,11-dienoic acid；(10E,12Z)-(9S)-9-Hydroxyoctadeca-10,12-dienoic acid
HIF-1 signaling pathway	11	2	ko04066	ncbi_692156(ENO3);ncbi_396859(NOS2);ncbi_396862(TIMP1);ncbi_397083(EGF);ncbi_397108(GP91-PHOX);ncbi_399500(IL6);ncbi_100626014(CAMK2A);ncbi_100518131(PIK3CD);ncbi_100158218(ENO4);ncbi_100522792(PRKCB);ncbi_100518663(PLCG2)	POS_M159T136;NEG_M157T90
Arginine biosynthesis	4	3	ko00220	ncbi_396859(NOS2);ncbi_397115(ARG1);ncbi_100521318(GPT2);ncbi_414411(ASS1)	POS_M174T105_1;POS_M175T87;POS_M176T93
Arachidonic acid metabolism	8	6	ko00590	ncbi_733634(AKR1C1);ncbi_767625(GPX2);ncbi_100512662(PLA2G2D);ncbi_100513737(PLA2G2F);ncbi_396971(ALOX15);ncbi_100514108(PLA2G5);ncbi_100519306(PLB1);ncbi_100514811(PLA2G4E)	POS_M335T821;POS_M339T801;POS_M353T769;POS_M353T613;NEG_M333T786;NEG_M351T793
Drug metabolism – other enzymes	8	1	ko00983	ncbi_397478(CES1);ncbi_100157205(RRM2B);ncbi_100510917(LOC100510917);ncbi_100624788(LOC100624788);ncbi_100522709(UPP2);ncbi_100625277(UPP1);ncbi_100316853(RRM2);MSTRG.15996(CES1)	POS_M133T154
Intestinal immune network for IgA production	10	2	ko04672	ncbi_100038026(TNFSF13B);ncbi_100135049(SLA-DOB);ncbi_100515419(CD28);ncbi_396659(CXCR4);ncbi_397315(PIGR);ncbi_100517087(TNFRSF17);ncbi_100526050(AICDA);ncbi_110260624(TNFRSF13C);MSTRG.15983(IGHA1);MSTRG.7693(HLA-DRB1)	9-cis-Retinoic acid

## Discussion

Since its first introduction in 1989 by Strachan et al. as a model in which non-hygienic contact between children and the environment was proposed to protect against allergic disease development (22), the hygiene hypothesis has been supported by a wealth of evidence highlighting the immunomodulatory properties of helminths and other microorganisms (23, 24). The immunological basis for the hygiene hypothesis remains incompletely understood but may be linked to the fact that the lack of early-life exposure to microorganisms can prevent regulatory T cell activation, thus promoting allergic disease development and autoimmunity (25). These results have been supported by studies of mice that either were or were not infected with gastrointestinal nematodes (*Heligmosomoides polygyrus*, *Trichuris muris*, or *Nippostrongylus brasiliensis*) and models of disease including peanut allergies and allergic disease. Similar analyses have also been conducted with murine models of collagen-induced arthritis or colitis to explore the relationship between microbial exposure and autoimmunity. In general, these studies have revealed that parasitic infections tend to protect against allergies and autoimmunity, reducing the number of cells in bronchoalveolar lavage fluid (BALF) with particular reductions in lymphocyte and eosinophil levels together with decreases in the levels of allergen-specific IgE in the serum and BALF together with declines in airway hyperreactivity (26–28). *H. polygyrus* infection also protected mice against the development of peanut-specific IgE upon immunization and prevented anaphylaxis when animals were subjected to peanut challenge (29). While these models are subject to some inherent limitations, they nonetheless provide support for the hygiene hypothesis.

While many studies have now offered evidence in favor of the hygiene hypothesis, many gaps in this model remain that necessitate detailed studies of the underlying immunological mechanisms. Accordingly, in this study Meishan piglets were used as an experimental model to compare how differences in rearing conditions (NC vs. ASF) would impact immune status. Many other metabolomic and transcriptomic studies of pigs exposed to environmental stressors have been published to date (11, 30). This study, however, is the first to our knowledge to conduct an integrated metabolomic and transcriptomic analysis in Meishan pigs, highlighting phenotypes that are dependent on rearing conditions. The observed changes in DEG and DAM levels are presumably linked to differences in immune status between these two groups owing to differences in metabolic gene regulation. Consistently, these genes and metabolites were enriched in the immune-related PPAR signaling and intestinal IgA production pathways, both of which are critically important for host resistance against pathogenic microbe infection (31, 32). Mechanistically, PPARγ can antagonize hepatic stellate cell activation in response to hypoxia through the regulation of PI3K/AKT and cGMP/PKG signaling activity (33). Exposure to a diverse range of microbes in the gastrointestinal tract can promote sustained B cell repertoire diversification and the T cell-independent production of antibodies, including high levels of IgA (34). These findings align well with prior studies demonstrating the value of testing immunological hypotheses in pigs given their status as large outbred mammals with many similarities to the human population, with 16S rRNA sequencing having successfully demonstrated that environmental exposures in the rearing environment can alter the diversity and quantity of the porcine ileal microbiota (35). Another study further highlighted clear differences in the composition of the ileal epithelial commensal microbiome when comparing pigs to indoor, outdoor, or indoor isolator-antibiotic conditions through 56 days of age (9), with the notable enrichment of the Firmicutes including beneficial Lactobacillaceae family members in pigs raised under outdoor conditions (77.2% of sequences) as compared to the indoor (12.8%) and isolator-antibiotic (3.58%) groups. In contrast, potentially pathogenic microbes were more common in the indoor groups and were absent in the outdoor group. These findings, together with the present results, thus support the hygiene hypothesis by suggesting that differences in rearing conditions can shape innate and adaptive immunity. However, future studies focused on the link between the intestinal microbiome and these immune responses will be needed to strengthen the evidence base for this model. Although this study revealed the immunological molecular mechanism of the “hygiene hypothesis” by using the transcriptome and metabolome, the human “hygiene hypothesis” believes that the less contact with pathogens in the early life, the greater the risk of immune mediated diseases in the later life, suggesting that the abundance of microorganisms in the growth environment can affect the development of the early immune system of animals, but the regulatory mechanism is still unclear. In the future, piglets need to be used as experimental animals to analyze the abundance of microorganisms in the growth environment and the regulatory role of early gut microbiota establishment and immune enhancement in pigs through metagenomic sequencing, laying the foundation for improving pig disease resistance through fecal transplantation in the future, and providing new basis for the immunological mechanism of the “hygiene hypothesis.”

## Conclusion

In summary, the present analyses highlighted several immune-related pathways that were differentially engaged in Meishan pigs reared under free-range conditions relative to those reared under controlled conditions based on transcriptomic and metabolomic profiles of duodenal samples from these animals. Specifically, these integrated metabolomic and transcriptomic analyses suggested that the PPAR signaling and IgA production pathways are differentially enriched between the NC and ASF groups, including key genes (*SCD, GK, LPL, FABP6, FADS2, PLIN4, FABP2, TNFRSF13C, TNFSF13B, IGHA1, PIGR*, *CXCR4, CD28, SLA-DOB,* and *AICDA*) and metabolites {(9Z,11E)-(13S)-13-Hydroxyoctadeca-9,11-dienoic acid; (10E,12Z)-(9S)-9-Hydroxyoctadeca-10,12-dienoic acid; 9-cis-Retinoic acid}, which will provide an important area of focus for future studies exploring the immunological basis for the hygiene hypothesis.

## Data availability statement

The data presented in the study are deposited in the GenBank Sequence Read Archive repository, accession number PRJNA998540.

## Ethics statement

The animal study was reviewed and approved by Animal Experiments Ethics Committee and Institutional Animal Care and Use Committee (IACUC) of Huaiyin Normal University. Written informed consent was obtained from the owners for the participation of their animals in this study.

## Author contributions

YL and GZ: conceptualization. YL and YS: methodology. SQ and ZZ: validation. JZ: formal analysis. QZ: investigation. PX: resources. LW and YL: data curation and visualization. YL: writing—original draft preparation, supervision, and funding acquisition. TQ: writing—review and editing. GZ: project administration. All authors contributed to the article and approved the submitted version.

## Funding

This work was supported by National Natural Science Foundation of China (31902127), Natural Science Foundation of Colleges of Jiangsu Province (19KJB230003), and Huai’an “Huai Shang Ying Cai” plan, the College Students’ Innovation and Entrepreneurship Training Program of Huaiyin Normal University (202210323032Z).

## Conflict of interest

The authors declare that the research was conducted in the absence of any commercial or financial relationships that could be construed as a potential conflict of interest.

## Publisher’s note

All claims expressed in this article are solely those of the authors and do not necessarily represent those of their affiliated organizations, or those of the publisher, the editors and the reviewers. Any product that may be evaluated in this article, or claim that may be made by its manufacturer, is not guaranteed or endorsed by the publisher.

## Footnotes

^1^http://metlin.scripps.edu

^2^https://mona.fiehnlab.ucdavis.edu/

^3^https://www.ncbi.nlm.nih.gov/genome/?term=pig

## Abbreviations

NC: normal captivity
ASF: arch soil free-range
DEGs: differentially expressed genes
DAMs: differentially abundant metabolites
FDR: false discovery rate
GO: gene ontology
KEGG: Kyoto Encyclopedia of Genes and Genome
POS: positive ion
NEG: negative ion
